# The Intravenous Injection of Medicaments and Its Importance in General Practice

**Published:** 1908-05

**Authors:** 


### The Intravenous Injection of Medicaments and Its Importance in General Practice.

EDWIN FRANCKE, Berlin. (*Medizinische Klinik,* January 5, 1908), says: in severe infections, sepsis, puerperal processes, collargol should be tried in 4 to 5 per cent. solution, 2 grams (30 grains), being injected intravenously every day or second day, according to the nature of the disease and the effect of the remedy. In one case of severe gonorrheal sepsis, with chills persisting for weeks, one such injection effected prompt and permanent defervescence.

Collargol injections are usually followed by a chill with temperature up to 41° C. (105 8° F.); but this phenomenon has no untoward consequences. The use of collargol by intravenous injection is superior to its introduction by enema or inunction, and should be resorted to more often than is now the case.

DR. ERNST RUNGE, Assistant at the University Gynecological

Clinic of the Charité, Berlin. (*Berliner klin. Wochenschrift*, March 16, 1908), says: in many cases of streptococcus infection, especially in pyemic processes, collargol intravenously (5 c.c. of a 5 per cent. solution daily for two or three days, injected into the cubital vein) occupies the first rank. The manner in which the silver introduced into the body is destructive of the streptococcus, has not yet been entirely cleared up.

Sellei and Unterberg, Budapest. (*Berl. klin. wochenschr.*, September 2, 1907), remark that lavage of the renal pelvis is indicated when internal treatment is unavailing. The authors have used collargol in 1 to 2 per cent. solutions, and noted good effects from this remedy.

